# Identification of Conserved Linear Epitopes on Viral Protein 2 of Foot-and-Mouth Disease Virus Serotype O by Monoclonal Antibodies 6F4.D11.B6 and 8D6.B9.C3

**DOI:** 10.3390/antib13030067

**Published:** 2024-08-07

**Authors:** Wantanee Tommeurd, Kanyarat Thueng-in, Sirin Theerawatanasirikul, Nongnaput Tuyapala, Sukontip Poonsuk, Nantawan Petcharat, Nattarat Thangthamniyom, Porntippa Lekcharoensuk

**Affiliations:** 1 Interdisciplinary Graduate Program in Genetic Engineering, The Graduate School, Kasetsart University, Bangkok 10900, Thailand; w.tommeurd@gmail.com (W.T.); fvetsrth@ku.ac.th (S.T.); 2 School of Pathology, Translational Medicine Program, Institute of Medicine, Suranaree University of Technology, Nakhon Ratchasima 30000, Thailand; kanyarat.th@sut.ac.th; 3 Department of Anatomy, Faculty of Veterinary Medicine, Kasetsart University, Bangkok 10900, Thailand; 4 Protein-Ligand Engineering and Molecular Biology Research Team, National Center for Genetic Engineering and Biotechnology (BIOTEC), National Science and Technology Development Agency (NSTDA), Pathum Thani 12120, Thailand; nongnaput.tuy@biotec.or.th; 5 Department of Pathology, Faculty of Veterinary Medicine, Kasetsart University, Bangkok 10900, Thailand; sukontip02@gmail.com; 6 Department of Microbiology and Immunology, Faculty of Veterinary Medicine, Kasetsart University, Bangkok 10900, Thailand; nantawan_ph@hotmail.com; 7 Research and Development Department, Animal Health and Diagnostic Center, CPF (Thailand) Public Company Limited, Bangkok 10530, Thailand; nattarat.tha@cpf.co.th

**Keywords:** foot-and-mouth disease virus, monoclonal antibody, hybridoma, phage display

## Abstract

Foot-and-mouth disease (FMD) is a highly infectious disease of cloven-hoofed animals with a significant economic impact. Early diagnosis and effective prevention and control could reduce the spread of the disease which could possibly minimize economic losses. Epitope characterization based on monoclonal antibodies provide essential information for developing diagnostic assays and vaccine designs. In this study, monoclonal antibodies raised against FMD virus (FMDV) were produced. Sixty-six monoclonal antibodies demonstrated strong reactivity and specificity to FMDV. The purified monoclonal antibodies were further used for bio-panning to select phage expressing specific epitopes from phage-displayed 12 mer-peptide library. The phage peptide sequences were analyzed using multiple sequence alignment and evaluated by peptide ELISA. Two hybridoma clones secreted monoclonal antibodies recognizing linear epitopes on VP2 of FMDV serotype O. The non-neutralizing monoclonal antibody 6F4.D11.B6 recognized the residues 67–78 on antigenic site 2 resinding in VP2, while the neutralizing monoclonal antibody 8D6.B9.C3 recognized a novel linear epitope encompassing residues 115–126 on VP2. This information and the FMDV-specific monoclonal antibodies provide valuable sources for further study and application in diagnosis, therapeutics and vaccine designs to strengthen the disease prevention and control measures.

## 1. Introduction

Foot-and-mouth disease (FMD) is one of the most infectious viral diseases of coven-hoofed animals with significant economic losses. The FMD virus (FMDV) is a single-stranded, positive-strand RNA virus which belongs to the genus *Aphthovirus* in the family *Picornaviridae*. The viral genome in 8.5 kb in length which upon translation is cleaved into four structural proteins and -eight non-structural proteins [1]. The four structural proteins, VP1, VP2, VP3 and VP4, are capsid proteins composed of immunodominant epitopes on the virion surface. FMDV is divided into seven serotypes including O, A, C, SAT 1, SAT 2, SAT 3, and Asia 1. The most frequent cause of global outbreaks is serotype O [2,3]. FMDV is highly genetically and antigenically variable, which each serotype composes of many topotypes and lineages [4], but there is no cross protection among serotypes. Therefore, immunity raised by one serotype cannot protect against future infections with the other serotypes and the antibodies against FMDV are mostly serotype specific.

FMDV is an RNA virus and its RNA-dependent RNA polymerase works without proofreading during virus replication, that leads to the emergence of novel genetic and antigenic variants of FMDV [5]. Due to the high mutation rates ranging from 10^−3^ to 10^−5^ nucleotides per site per genome replication, FMDV exists as quasispecies in nature. The viral population is not genetically 100% identical but rather a combination of highly related sequences. This characteristic allows adaptation of the virus swarm that could replicate in various specific environments [6]. Although antigenic variation within FMDV occurs frequently, the changes are restricted to specific regions of the viral capsid proteins since changes in other regions would destroy the structural integrity of virus particles [7].

The development of rapid diagnostic tools is crucial for the effective control and eradication of infectious diseases. The clinical symptoms caused by FMDV infection are difficult to distinguish from other vesicular diseases [8]. Furthermore, the clinical signs vary significantly among different species and individual animals [9]. Therefore, early and accurate diagnosis is a key factor for preventing the transmission of the virus from an affected farm to other herds, particularly in the absence of a universal vaccine for all FMDV serotypes. In addition, the quasispecies nature of FMDV leads to the generation of viral populations with distinct antigenic properties, and therefore it poses challenges for the development of diagnostic tools. In most cases, monoclonal antibodies are essential precursors required to improve the performance of diagnostic assays such as ELISA or chromatographic strip tests.

The phage display technique is widely used for epitope identification due to its cost-effectiveness and rapidity. The technique is based on the direct linkage between the genotype and phenotype of bacteriophages, in which a genetic modification of phage DNA combined with a gene encoding peptide is able to display a peptide on the surface of the bacteriophage. In the phage display technique, panning of the random peptide libraries against a specified target can identify mimotopes, which mimic the particular epitope structures, but are not completely identical to the corresponding epitopes, yet can elicit the specific antibody responses to their cognate epitopes [10]. Previously, Wang and colleagues [11] used the phage display techniques to identify the epitopes recognized by monoclonal antibodies raised against different serotypes of FMDV. In this approach, a monoclonal antibody was immobilized on the solid surface for bio-panning against peptide libraries. They successfully identified a neutralizing epitope with the amino acid sequence ^141^SXRGXLXXLXRR^152^, on the G-H loop of the VP1 protein of FMDV serotype Asia1. In another study, the consensus motif GDLNVRT was identified as a linear neutralizing epitope in the highly conserved residues of the VP1 protein of FMDV serotype O using a 12-mer random peptide phage display library [12], whereas a consensus motif ETTXLE which was closed to conserved residues at the N-terminus of the VP2 protein of FMDV was also characterized [13]. Additionally, the consensus motif YxxPxGDLG, which was highly homologous to the ^135^YxxPxxxxxGDLG^147^ on the capsid protein VP1 of FMDV serotype A, was identified using a randomized 12-mer phage-displayed peptide library [14].

In this study, we describe the generation and characterization of two monoclonal antibodies, 6F4.D11.B6 and 8D6.B9.C3, against FMDV type O O/TAI/189/1987. The linear epitopes recognized by these two monoclonal antibodies were identified using a random phage-displayed 12-mer peptide library.

## 2. Materials and Methods

### 2.1. Cells and Viruses

Baby Hamster Kidney 21 (BHK21) cells (ATCC, Manassas, VA, USA) were maintained in Minimum Essential Medium (MEM) with 40 mM L-glutamine and 1% antibiotic-antimycotic. All reagents were purchased from Gibco, Thermo Fisher Scientific, Waltham, MA, USA. Virus isolates used in this study were FMDV serotypes O and A collected from a swine farm in western Thailand in different years including O/TAI/SEA/Mya98/CB25/2019 and A/TAI/ASIA/Sea97/NP05/2019. Inactivated FMDV O/TAI/189/1987 used for mice immunization was contributed by the Bureau of Veterinary Biologics, DLD, Nakhon Ratchasima, Thailand.

### 2.2. Preparation of Monoclonal Antibodies

Six four-week-old female BALB/c mice (National Laboratory Animal Center, Nakhon Pathom, Thailand) were divided into a control group and an immunized group. FMDV serotype O antigen was purified by PEG precipitation and 15–45% sucrose density gradient ultracentrifugation [15]. Mice were injected subcutaneously with 10 µg of purified FMDV serotype O antigen and an equal volume of complete Freund’s adjuvant (Sigma-Aldrich, St. Louis, MO, USA) for the first immunization and incomplete Freund’s adjuvant (Sigma-Aldrich, St. Louis, MO, USA) for the second and third immunizations at 3-week intervals. The control group was immunized with PBS mixed with complete Freund’s adjuvant at the ratio of 1:1. At day 7 after the last immunization, blood was collected to determine the titer of FMDV serotype O-specific antibodies. Subsequently, the mice were immunized intravenously with non-adjuvanted antigen. At day 3 after the final booster, the spleens of immunized mice were collected and fused with myeloma cells (P3-X63-Ag8.653 cells) in the media containing 50% (*w*/*v*) polyethylene glycol (Sigma-Aldrich, St. Louis, MO, USA) following the published protocol [16]. The fused cells were cultured in 96-well plates at 1 × 10^5^ cells/well in HAT selective medium (Corning, Corning, NY, USA). After incubation at 37 °C for 1 week, the viable hybridoma cells were observed under an inverted microscope. The culture supernatant of hybridoma cells at 70–80% confluence was collected and screened for the production of antibody by indirect enzyme-linked immunosorbent assay (ELISA). The positive antibody-producing hybridoma were repeatedly cloned for three times by the limiting dilution method [16]. The monoclonal antibodies were purified using Amintra™ Protein-G resin (Expedeon, Cambridge, UK).

### 2.3. Production of the Recombinant VP Capsid Proteins

The nucleotide sequences of the VP1, VP2, and VP3 capsid proteins of FMDV serotype O (O189) [17] were used as the template for the polymerase chain reaction with gene specific primers (Table S1). The PCR products were cloned into the *Bam*HI/*Hind*III restriction sites of the prokaryotic expression vector pQE-80L (QIAGEN, Hilden, Germany). Subsequently, the recombinant plasmids were transformed into *E. coli* BL21 (DE3) cells, and expression of the recombinant proteins was induced using 0.2 mM IPTG (Sigma-Aldrich, St. Louis, MO, USA) for 16 h. The protein expression was verified by SDS-PAGE analysis using a 12% gel, according to the standard procedure [18]. The expressed VP proteins were purified by affinity chromatography based on histidine tag, using Protino Ni-TED Resin-gravity-flow column (Macherey-Nagel, Düren, Germany), according to the manufacturer’s instruction.

### 2.4. Indirect Enzyme-Linked Immunosorbent Assay (ELISA)

ELISA was modified from the previously published protocol [19]. FMDV serotype O antigen, 1 µg/mL, was coated onto each well of 96-well plates. The coated antigen was removed, and the available sites in each well were blocked with 5% skim milk (Difco Laboratories, Detroit, MI, USA) at 37 °C for 1 h. Each well was washed three times with PBS containing 0.05% Tween 20 (PBST). Then, 100 µL of the hybridoma culture medium was added into each well before incubating at 37 °C for 1 h. The wells were washed with PBST for three times, and then incubated with goat anti-mouse IgG conjugated with HRP (Sigma-Aldrich, St. Louis, MO, USA) diluted 1:3000 in PBST at 37 °C for 1 h. The excess antibody was washed three times, and the reaction was incubated with ABTS substrate (Thermo Scientific, Waltham, MA, USA). Finally, the absorbance was measured at a wavelength 405 nm using BioTek Microplate Readers (Agilent Technologies, Santa Clara, CA, USA).

### 2.5. Immunoperoxidase Monolayer Assay (IPMA)

BHK-21 cells in a 96-well plate were inoculated with 1 × 10^3^ TCID_50_ of each FMDV and incubated at 37 °C for 24 h. IPMA was performed as described previously [20]. Cold methanol was used for fixing the infected cells at room temperature for 30 min, followed by incubating with 1% H_2_O_2_ (Sigma-Aldrich, St. Louis, MO, USA) in PBS for 50 min. The hybridoma culture supernatants, diluted 1:10 and 1:100 in PBST, were added to the plate and incubated at 37 °C for 1 h. Subsequently, the plate was incubated with Protein G conjugated with HRP (Sigma-Aldrich, St. Louis, MO, USA) diluted 1:500 in PBST. After each step, the plate was washed with PBST five times. Lastly, the reaction was incubated with DAB substrate solution (Agilent Technologies, Santa Clara, CA, USA) before rinsing with plenty of deionized water to stop the reaction. The antibody-antigen reactivity was examined under an inverted microscope (Olympus, CK X4, Tokyo, Japan).

### 2.6. Western Blot

The monoclonal antibody profiles were investigated by western blot following the previously published protocol [21]. FMDV serotype O antigens and the recombinant proteins were electrophoresed through 12% SDS-PAGE (Thermo Scientific, Waltham, MA, USA), which was subsequently blotted onto a nitrocellulose membrane (Bio-Rad Laboratories, Hercules, CA, USA) in Tris-Glycine transfer buffer (25 mM Tris, 192 mM glycine and 20% Methanol, pH 9.9) using the wet transfer method. After blocking with 5% skim milk (Difco Laboratories, Detroit, MI, USA), the blotted membrane was soaked in the hybridoma culture supernatants or monoclonal antibodies at room temperature for 1 h and incubated with HRP-conjugated goat anti-mouse IgG (Sigma-Aldrich, St. Louis, MO, USA) diluted at 1:2000 in TBST (50 mM Tris-HCl pH 7.5, 100 mM NaCl, 1 mM EDTA, 0.1% Tween 20). After each step, the membrane was washed with TBST three times. Finally, the membrane was incubated in DAB substrate solution (Agilent Technologies, Santa Clara, CA, USA) until the color developed. The reaction was stopped by rinsing the membrane with sufficient deionized water.

### 2.7. Micro-Neutralization Assay

One hundred and fifty microliters of 1.5 µg of the purified anti-FMDV monoclonal antibodies in MEM was incubated with 150 µL of 100 TCID_50_ of each FMDV isolate (O/TAI/189/1987 and O/TAI/SEA/Mya98/CB25/2019) at 37 °C for 1 h. The mixtures were 10-fold serially diluted from 10^−1^ to 10^−5^ before adding into each well of the 96-well plate (50 µL/well) withfour wells per dilution. Then, 100 µL of 3 × 10^4^ cells of BHK-21 cell-suspension were added to each well before incubating at 37 °C for 48 h. The cytopathic effect was examined under an inverted microscope. Neutralizing antibody titer is the reciprocal of the highest antibody dilution that completely inhibits 100 TCID_50_ of the virus.

### 2.8. Bio-Panning

The Ph.D.-12™ phage display peptide library (New England Biolabs, Ipswich, MA, USA) was employed for epitope identification. The affinity selection of the phage clones from the random peptide library was performed following the manufacturer’s instructions. Bio-panning was conducted by coating 2 µg/mL (300 ng/well) of purified anti-FMDV monoclonal antibody clones 6F4.D11.B6 and 8D6.B9.C3 (in house preparation), followed by blocking with 5 mg/mL BSA (Sigma-Aldrich, St. Louis, MO, USA) in coating buffer (0.1 M NaHCO_3_, pH 8.6). After washing with TBST, the diluted phage at a final concentration of 2 × 10^11^ pfu/mL was added to each well and incubated at room temperature for 1 h. The unbound phages were washed with TBST ten times. Subsequently, the bound phages were eluted with elution buffer (0.2 M glycine-HCl, pH 2.2), and then the eluates were neutralized with 1 M Tris-HCl, pH 9.1. Thereafter, the phage eluates were titrated and amplified in *E. coli* ER2738 (New England Biolabs, Ipswich, MA, USA) cultured on LB agar containing 0.05 mg/mL IPTG (Sigma-Aldrich, St. Louis, MO, USA), and 0.04 mg/mL Xgal (Sigma-Aldrich, St. Louis, MO, USA). In addition, the phage eluates were also amplified in the *E. coli* ER2738 for using as a phage stock in the next round of bio-panning. After three rounds of bio-panning, the blue plaques were collected from the titration plate and amplified in the *E. coli* ER2738 (New England Biolabs, Ipswich, MA, USA). The amplified phages were purified using 20% PEG (PEG8000, Sigma-Aldrich, St. Louis, MO, USA) in 2.5 M NaCl, and then the DNA was extracted using sodium iodide (Sigma-Aldrich, St. Louis, MO, USA). The extracted DNA of each isolated phage was used as the template for PCR amplification with pIII M13 gene specific primers designed based on the sequence provided in the Ph.D.-12 Phage Display Peptide Library Kit (New England Biolabs, Ipswich, MA, USA). The sequences of forward and reverse primers were 5′-TTATTCGCAATTCCTTTAG-3′ and 5′-CCTCATAGTTAGCGTAACG-3′, respectively. The PCR products were cloned into pGEM-T easy vector (Promega, Madison, WI, USA) and transformed into *E. coli* DH5α. Eight to nine phage clones of the recombinant pGEM-T plasmids were isolated and sequenced (Macrogen, Seoul, Republic of Korea). The sequences of the selected phage clones were verified by DNA analysis (Version Lasergene 11, DNASTAR Lasergene, Madison, WI, USA). The phage peptide sequences were aligned with amino acid sequences of viral protein of FMDV serotype O using Clustal Omega program available in Jalview software (Version 2.11.2.6) as described elsewhere [22].

### 2.9. Phage ELISA

To investigate the reactivity of monoclonal antibodies and individual phage clones, a phage ELISA was performed, as described in the manufacturer’s instruction (New England Biolabs, Ipswich, MA, USA). Briefly, a 96-well plate (Corning, Corning, NY, USA) was coated with the purified anti-FMDV monoclonal antibodies (1 µg/well) at 4 °C overnight. The purified phage clones were incubated with the coated monoclonal antibody at room temperature for 1 h. After washing, the bound phage was incubated with HRP-conjugated anti-M13 phage antibody (Sino Biological, Beijing, China) at a dilution of 1:5000 at room temperature for 1 h. Finally, the reaction was incubated with ABTS substrate (Thermo Scientific, Waltham, MA, USA) at 37 °C for 30 min, and the absorbance was measured at wavelength 405 nm using BioTek Microplate Readers (Agilent Technologies, Santa Clara, CA, USA). A monoclonal antibody losing the ability to bind FMDV antigens (4F10.C4.D10) and BSA were used as the negative controls.

### 2.10. Peptide ELISA

To confirm whether the monoclonal antibodies could bind specifically to the phage peptide sequences, a peptide ELISA was conducted. The selected phage peptides were synthesized (Cellmano Biotech, Hefei, China), with over 95% purity. The synthetic peptides were diluted in PBS to the final concentrations of 5 µg/mL and coated onto a 96-well plate. The empty sites in each well were blocked with 5% skim milk (Difco Laboratories, Detroit, MI, USA). Afterwards, each monoclonal antibody at the concentration of 50 µg/mL was added into the wells, 100 µL/well, followed by incubating at 37 °C for 1 h. After washing, the bound monoclonal antibody was incubated with HRP-conjugated goat anti-mouse IgG (Sigma-Aldrich, St. Louis, MO, USA) diluted 1:2000 in PBST. After each incubation step, the wells were washed with PBST three times. Lastly, the enzyme reaction was incubated with ABTS substrate (Thermo Scientific, Waltham, MA, USA) for 30 min, and the absorbance was measured at wavelength 405 nm using BioTek Microplate Readers (Agilent Technologies, Santa Clara, CA, USA).

### 2.11. Statistical Analysis

A statistical analysis was performed by the statistical software Graphpad Prism (Version 10.2.2) (San Diego, CA, USA). Data were analyzed using one way ANOVA and considered statistically significant when *p* < 0.05.

### 2.12. PCR Amplification of Immunoglobulin Light and Heavy Chain Variable Genes

Total RNA from monoclonal antibody-producing hybridoma cell lines was isolated using TRIzol™ Reagent (Invitrogen, Carlsbad, CA, USA) according to the manufacturer’s protocol. The RNA pellet was resuspended in DNase/RNase-Free Distilled Water (Invitrogen, Carlsbad, CA, USA). cDNA was synthesized using random primer and SuperScript™ III First-Strand Synthesis System (Invitrogen, Carlsbad, CA, USA) according to the oligo dT protocol provided by the manufacturer and used as the template for the following PCR.

PCR reactions were carried out using Taq DNA polymerase (Thermo Scientific, Waltham, MA, USA). For the 10 µL reaction, it contained 5 pmol of forward and reverse primers [23], 0.1 µL of Taq polymerase, 0.2 µL of 10 mM dNTPs, 0.2 µL of 5X HF buffer, and 1 µL of the cDNA template. The PCR conditions were: 98 °C for 2 min, 35 cycles of 98 °C for 10 s, 64 °C for 15 s and 72 °C for 30 s, and a final incubation at 72 °C for 10 min. The GeneJET PCR Purification kit (Thermo Scientific, Waltham, MA, USA) was used for cleaning up the PCR products. Subsequently, A-tailing with Taq DNA polymerase (Thermo Scientific, Waltham, MA, USA) was performed according to the manufacturers’ instructions. The 50-µL A-tailing reaction contained 1 µL of dATP, 5 µL of 10X (NH_4_)_2_SO_4_, 3 µL of 25 mM MgCl_2_, 0.2 µL of Taq DNA polymerase, and 30 µL of the PCR products. The reaction was incubated at 72 °C for 20 min. The A-tailing products were analyzed by 1.2% agarose gel electrophoresis. DNA bands of expected sizes were gel extracted by GeneJET Gel Extraction Kit (Thermo Scientific, Waltham, MA, USA) before ligated into pGEM-T easy vector (Promega, Madison, WI, USA). The ligation reaction was transformed into *E. coli* DH5α (Invitrogen, Carlsbad, CA, USA). The GeneJET Plasmid Miniprep kit (Thermo Scientific, Waltham, MA, USA) was used for isolating the recombinant pGEM-T plasmids with insert and the variable genes were verified by DNA sequencing (Macrogen, Seoul, Republic of Korea).

### 2.13. Molecular Modeling of FMDV VPs and Monoclonal Antibodies

Molecular modeling was conducted utilizing the SWISS-MODEL server (https://swissmodel.expasy.org/, accessed on 28 June 2023) [24]. Advanced homology modeling techniques were employed to generate three-dimensional protein models based on template structures available in the Protein Data Bank (PDB) database. These models were constructed by considering sequence similarity and other relevant criteria. The amino acid sequence of the viral protein was based on VP2 DNA sequence of FMDV serotype O, O/TAI/189/1997 (O189), (property of the Bureau of Veterinary Biologics, DLD, Nakhon Ratchasima, Thailand). The template structures with the highest sequence identity (98.6%) to the target sequences, identified as PDB code ID: 1bbt.pdb [25], were selected for subsequent model construction. Additionally, the DNA sequences of immunoglobulin light and heavy chain variable regions of the monoclonal antibodies 6F4.D11.B6 and 8D6.B9.C3, comprising both light and heavy chains, were modeled using the same procedure. The deduced amino acid sequence of 6F4.D11.B6 was found to match PDB code ID: 6tcs.pdb [26], while the sequence of 8D6.B9.C3 was most identical to PDB code ID: 7det.pdb [27].

To ensure the robustness and reliability of the generated protein models, a rigorous structural evaluation was performed employing well-established criteria. MolProbity [28], QMEANDisCo [29], and Ramachandran plot analysis [30] were utilized to assess various aspects of the models, including their overall quality, stereochemical properties, and backbone torsion angles. Subsequently, the critical complementarity-determining regions (CDR1, CDR2, and CDR3) were predicted using the advanced algorithms of SCALOP [31] and Lyra [32], both accessed on 28 June 2023. All models were visualized and analyzed using Discovery Studio Visualizer, version 2021 (BIOVIA, Dassault Systèmes, San Diego, CA, USA), and UCSF Chimera, version 1.16 (UCSF, San Francisco, CA, USA) [33], to gain insights into their structural features and potential functional implications.

### 2.14. Molecular Docking to Predict FMDV VP2 Capsid Protein and Monoclonal Antibody Interactions

The protein-protein docking analysis was performed using the HADDOCK web server (https://rascar.science.uu.nl/haddock2.4/, accessed on 28 June 2023) [34,35] to investigate the interaction between the monoclonal antibody and the target protein, VP2. The crystal structure of VP2 was prepared by eliminating any bound ligands or solvent molecules and assigning appropriate protonation states. The identification of active residues on VP2 was guided by our experimental data, with particular emphasis on the predicted complementarity-determining regions (CDR1, CDR2, and CDR3) of each monoclonal antibody, which play a pivotal role in antigen recognition and binding. These active residues on VP2 were selected as potential binding sites for the monoclonal antibody.

The protein-protein docking calculations were executed using the HADDOCK scoring function, which integrates intermolecular energy terms and restraints derived from the active residues defined on VP2 and the CDR regions of the antibody. The resultant docking solutions were systematically analyzed to identify the most favorable docking poses and assess the binding affinity between monoclonal antibodies and VP2. For in-depth analysis and interpretation, the output structures were further examined and visualized using Discovery Studio Visualizer, version 2021 (BIOVIA, Dassault Systèmes, San Diego, CA, USA), and UCSF Chimera, version 1.16 (UCSF, San Francisco, CA, USA).

## 3. Results

### 3.1. Generation of Monoclonal Antibody

**Figure 1:**
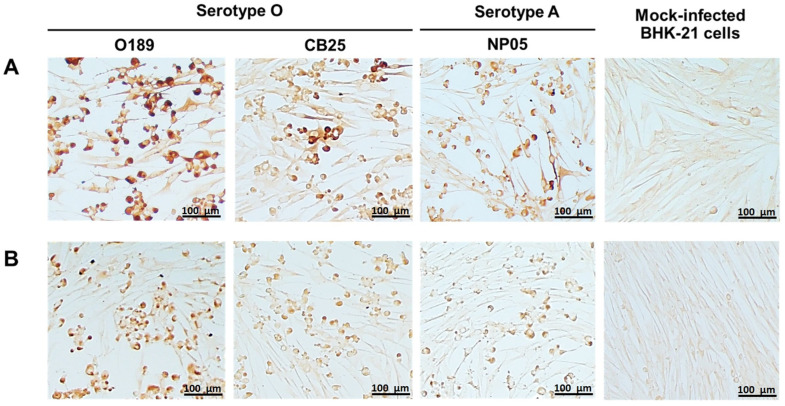
Reactivities of 6F4.D11.B6 and 8D6.B9.C3 to different strains of FMDV serotypes O and A in IPMA. BHK-21 cells infected with FMDV serotypes O, O/TAI/189/1987 (O189) and O/TAI/SEA/Mya98/CB25/2019 (CB25), and serotype A, A/TAI/ASIA/Sea97/NP05/2019 (NP05) were incubated with monoclonal antibodies (**A**) 6F4.D11.B6 and (**B**) 8D6.B9.C3. The specific reactivities between viral antigens and monoclonal antibodies were detected by IPMA. Mock-infected BHK-21 cells were used as a negative control.

Monoclonal antibodies against FMDV were produced by hybridoma cell lines generated from the fusion between the splenocytes from FMDV serotype O-immunized mice and murine myeloma cells. Single clonal hybridomas were selected by limiting dilution method three times. Supernatants from the hybridoma clones were screened for their reactivity and specificity to the partially purified FMDV serotype O (O189) by indirect ELISA, resulting in 66 FMDV-specific clones. Immunogenic characteristics of monoclonal antibodies from the 66 hybridoma clones were further investigated by western blot and IPMA. Among them, 6F4.D11.B6 and 8D6.B9.C3 demonstrated high affinity to the viral antigen in the indirect ELISA, IPMA, and western blot. After freezing and thawing, the affinity-purified monoclonal antibodies, 6F4.D11.B6 and 8D6.B9.C3, were re-tested by IPMA against FMDV serotype O (O189 and CB25) and serotype A (NP05). The results showed that both monoclonal antibodies could bind well to the FMDV antigens of both serotypes, and reacted strongly with O189 used to immunize mice (Figure 1).

**Figure 2:**
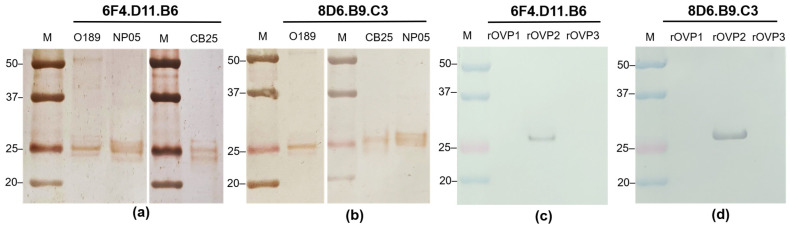
Immunoreactivity of the affinity-purified monoclonal antibodies 6F4.D11.B6 and 8D6.B9.C3 with FMDV antigens examined by western blotting. Monoclonal antibodies (**a**) 6F4.D11.B6 and (**b**) 8D6.B9.C3 were allowed to react with the blotted proteins from FMDV serotypes O, O/TAI/189/1987 (O189) and O/TAI/SEA/Mya98/CB25/2019 (CB25), and serotype A, A/TAI/ASIA/Sea97/NP05/2019 (NP05). In addition, immunoreactivities of the purified monoclonal antibodies (**c**) 6F4.D11.B6, and (**d**) 8D6.B9.C3 were observed with the recombinant viral proteins of FMDV O189 (rOVP1, rOVP2 and rOVP3). Both monoclonal antibodies reacted strongly with FMDV VP2.

In addition, the immunoreactivity of the purified monoclonal antibodies was investigated by western blotting with FMDV serotypes O and A. The results showed that the monoclonal antibodies 6F4.D11.B6 and 8D6.B9.C3 reacted strongly to proteins with molecular weight of approximately 25 kDa (Figure 2a,b), which is close to the sizes of FMDV capsid proteins VP1 (28 kDa), VP2 (26 kDa), and VP3 (27 kDa). The positive signals on the western blots generated by these monoclonal antibodies under reducing conditions indicated that they possibly recognized linear epitopes of the FMDV antigens. To reveal the viral proteins recognized by each monoclonal antibody, the affinity purified recombinant FMDV O189 VP1, VP2, and VP3 were used as antigens for testing specific reactivities in the western blot analysis. As shown in Figure 2c,d, monoclonal antibodies 6F4.D11.B6 and 8D6.B9.C3 exhibited strong signals with the recombinant VP2 capsid proteins. All complete western blot images were depicted in Figure S1.

The neutralizing activities of the monoclonal antibodies 6F4.D11.B6 and 8D6.B9.C3 were determined by a micro-neutralization assay on BHK-21 cells using two FMDV serotypes O, O/TAI/189/1987 (O189) and O/TAI/SEA/Mya98/CB25/2019 (CB25). The neutralizing titer of 8D6.B9.C3 against 100 TCID50 of both FMDV O189 and CB25 were 1:200 while that of 6F4.D11.B6 showed no neutralizing activity against the viruses.

### 3.2. Epitope Mapping by Phage Display Technique

To identify the epitopes recognized by the FMDV-specific monoclonal antibodies 6F4.D11.B6 and 8D6.B9.C3, bio-panning of 12-mer phage peptide library against these monoclonal antibodies was conducted. After three rounds of bio-panning, twenty individual plaques of phage clones bound to either monoclonal antibodies 6F4.D11.B6 or 8D6.B9.C3 were randomly picked from an *E. coli* lawn for phage amplification. DNA from these amplified phage clones was isolated and used as the template for PCR. Due to the presence of a 12-mer peptide on the pIII coating protein of M13 phage, pIII M13 gene-specific primers were designed to amplify the peptide sequence. The amplicons of pIII gene with the peptide inserts were cloned into plasmids and verified by nucleotide sequencing. After sequence analysis, some individual phages were discarded because of incorrect sequence. Only nine and eight individual phage sequences from 6F4.D11.B6 and 8D6.B9.C3 (Table 1) were further analyzed, respectively. Mimotope DNA sequences of seven out of nine phage clones obtained from the bio-panning against 6F4.D11.B6 were ‘HEWNRISDLSYA’ (Table 1). For 8D6.B9.C3, the sequence ‘FKQDAWEAVDIR’ was found in four out of eight clones, while the sequences ‘YFPVFPQFNVIQ’, ‘TLHGCCYNSMQR’, ‘AISPSRYFYDET’ and ‘SYSGGILSALTE’ were found once each in the remaining clones (Table 1). Additionally, the specificity of the selected phage clones with both monoclonal antibodies was further evaluated by phage ELISA. As shown in Table 1, the reactivities of all identified phage clones were higher than those of the negative wells (OD = 0.076) suggesting high specificity and affinity of these peptides for the monoclonal antibodies.

**Figure 3:**
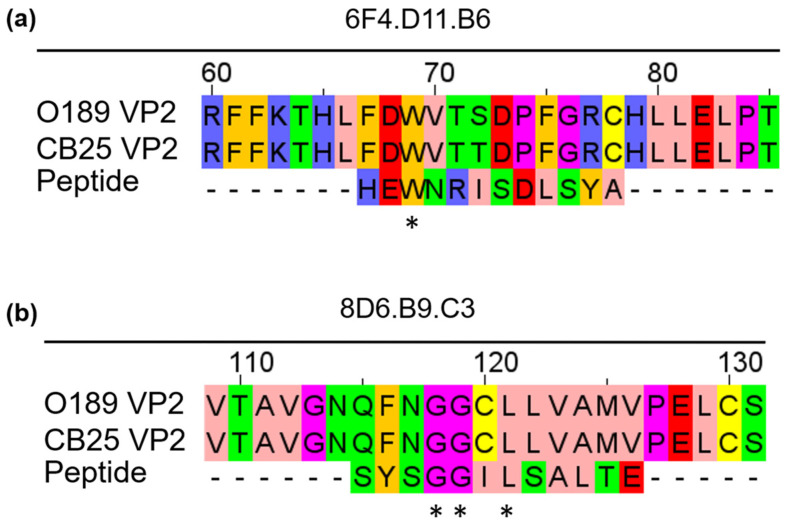
Alignment between amino acid sequences of phage peptides selected by bio-panning with FMDV-specific monoclonal antibodies and those of VP2 capsid protein from different FMDV serotype O isolates. (**a**) 6F4.D11.B6-specific phage peptide sequence ‘HEWNRISDLSYA’ and (**b**) 8D6.B9.C3-specific phage peptide sequence ‘SYSGGILSALTE’ were aligned with VP2 amino acid sequences. Each amino acid residue was colored based on the Zappos scheme, according to their physicochemical properties. Pink: aliphatic/hydrophobic, Amber: aromatic, Blue: positive charged, Red: negative charged, Green: hydrophilic, Magenta: proline and glycine, Yellow: cysteine. ‘*’ indicates identical amino acid residues between the VP2 and peptide.

We further aligned the phage peptide sequence ‘HEWNRISDLSYA’, which was found most frequently and demonstrated the highest specificity to 6F4.D11.B6 in the phage ELISA (Table 1) with amino acid sequences of the VP2 capsid protein from different strains of FMDV serotype O (O/TAI/189/1987, and O/TAI/SEA/Mya98/CB25/2019) using Clustal Omega, a multiple sequence alignment program. The analysis aligned the phage peptide sequence ‘HEWNRISDLSYA’ with the amino acid sequence ^67^FDWVTXDXFGRX^78^ of the VP2 of FMDV serotype O (Figure 3a). On the other hand, all five phage peptides obtained from 8D6.B9.C3 exhibited high specificity to the monoclonal antibody in the phage ELISA; therefore, each peptide was aligned with the amino acid sequences of the VP2 from FMDV serotype O (Figure S2). The analysis revealed that the alignment of the phage peptide sequence ‘SYSGGILSALTE’ with VP2 showed the highest percent sequence identity with no gap and contained the highest number of amino acid residues with conserved physicochemical properties in the alignment [36]. The phage peptide sequence ‘SYSGGILSALTE’ corresponded to the sequence ^115^QFNGGCLLVAMVP^126^ on the VP2 protein of FMDV serotype O (Figure 3b).

### 3.3. Validation of the Predicted Epitope by Peptide ELISA

**Figure 4:**
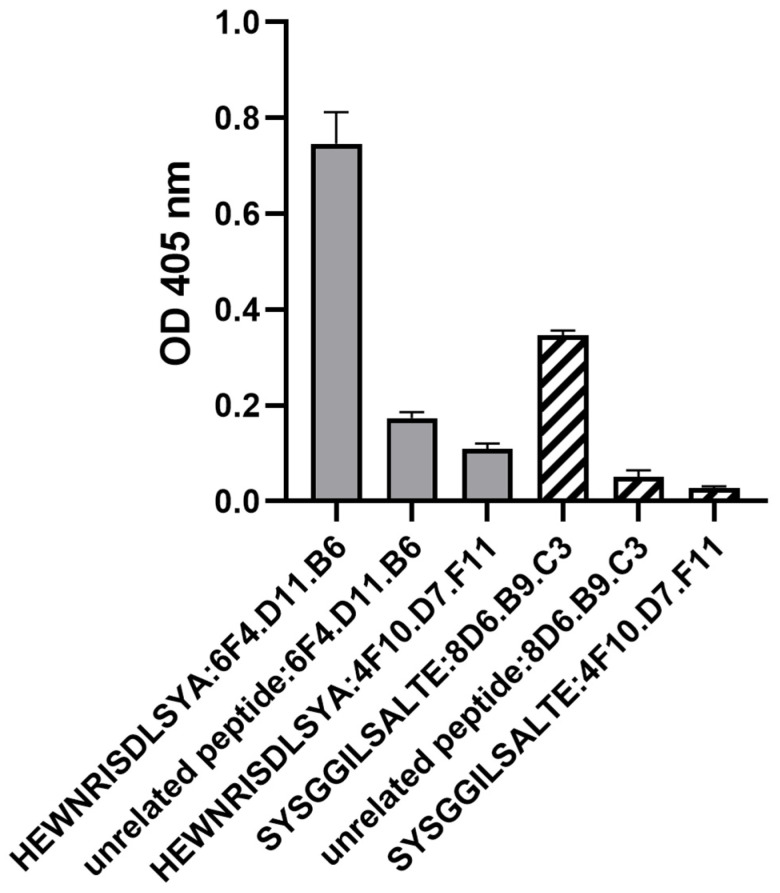
The binding specificity of the monoclonal antibodies and the corresponding synthetic peptides as evaluated by peptide ELISA. The monoclonal antibodies were allowed to bind to the coated peptides (5 µg/mL). The peptides ‘HEWNRISDLSYA’, and ’SYSGGILSALTE’ were selected by bio-panning with monoclonal antibodies 6F4.D11.B6 and 8D6.B9.C3, respectively. 4F10.D7.F11 is a negative monoclonal antibody control while an unrelated peptide is a negative peptide control. All data were obtained independently in triplicate, and are presented as the mean ± SEM of the triplicate experiments.

The peptides ‘HEWNRISDLSYA’ and ‘SYSGGILSALTE’ were synthesized and examined using indirect ELISA to validate their reactivity with 6F4.D11.B6 and 8D6.B9.C3, respectively. Monoclonal antibody 4F10.D7.F11 was included as a negative monoclonal antibody control because it had lost the ability to bind to FMDV antigens during the freezing and thawing process. The results showed that 6F4.D11.B6 and 8D6.B9.C3 could bind to their cognate peptides with high affinity, compared to those of the negative monoclonal antibody control (4F10.D7.F11) and an unrelated peptide (Figure 4; Table S2).

### 3.4. Prediction of the Interaction of FMDV Serotype O Capsid Protein VP2 and Monoclonal Antibodies by Molecular Docking Analysis

To predict the interactions between VP2 and monoclonal antibodies, 6F4.B11.D6 and 8D6.B9.C3, the immunoglobulin heavy and light chain variable genes of each monoclonal antibody were sequenced. These sequences were analyzed and translated to amino acids that were used for the molecular modeling. The generated 3D models of FMDV serotype O VP2 and monoclonal antibodies, 6F4.D11.B6 and 8D6.B9.C3, were compared to the template structures using the QMEANDisCo global score. Furthermore, the models were assessed using MolProbity scores and Ramachandran plots (Figures S3 and S4).

**Figure 5:**
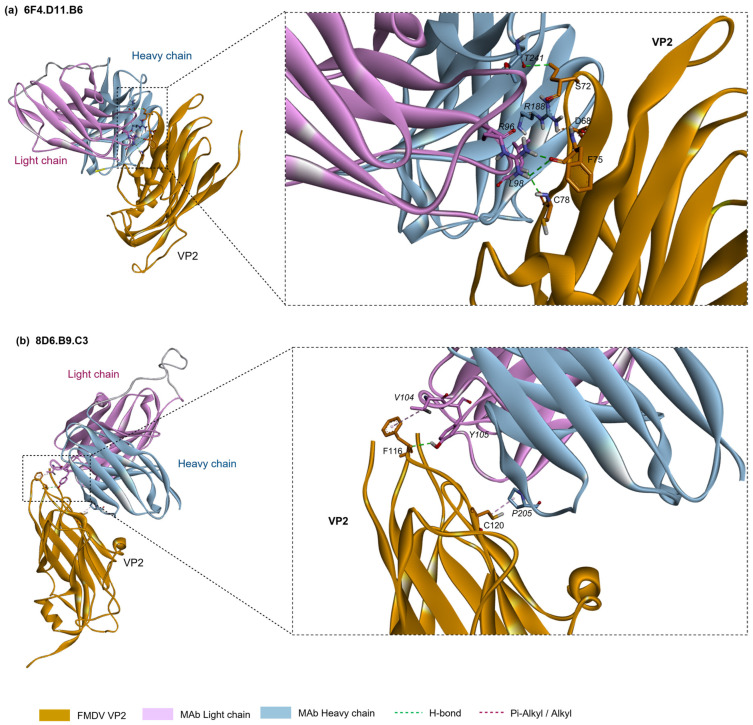
The interaction between FMDV serotype O capsid protein VP2 and monoclonal antibodies: (**a**) 6F4.D11.B6, (**b**) 8D6.B9.C3.

The molecular docking predictions were consistent with the mimotopes identified by the phage display technique in showing that the monoclonal antibodies 6F4.B11.D6 and 8D6.B9.C3 interacted with residues 67–78 and 115–126 on VP2 of FMDV serotype O, respectively. The prediction models of VP2 and 6F4.B11.D6 indicated that the side chains of the residues D68 and S72 of VP2 formed hydrogen bonds with the side chain atoms of R188 and T241 in the HCDR2 and HCDR3, respectively, while the residues F75 and C78 side chains also interacted with the side chains of R96 and L98 in the CDR3-light chain via hydrogen bonds. In addition, the docking between VP2 and 8D6.B9.C3 showed that the side chain atoms of the residues F116 and C120 in the DE loop of VP2 contacted the side chains of V104 at the HCDR3 and that of P205 at the LCDR2, respectively. The interactions between the structural proteins VP2 and monoclonal antibodies is depicted in Figure 5, while the CDR regions and the predicted interactive amino acid residues are shown in Figure S5.

## 4. Discussion

Monoclonal antibodies are widely utilized as advantageous biomolecules for a variety of applications, including infectious disease research, diagnostics and therapeutics. The development of diagnostic tests and vaccines for FMDV is crucial for disease control. Monoclonal antibodies specific to FMDV serotype O were first identified by McCullough and Butcher [37]. Subsequently, monoclonal antibodies against other serotypes were generated [38,39,40,41,42,43]. In 2007, Yang and his colleagues [44] reported two monoclonal antibodies, F1412SA and F21140SO, recognizing all seven serotypes of FMDV. In subsequent studies, specific epitopes recognized by monoclonal antibodies specific to FMDV were identified for further applications, such as neutralizing epitope identification for vaccine design [11,12,45] and developing diagnostic techniques, including a double antibody sandwich ELISA using two monoclonal antibodies [46] and a multiple lateral flow immunochromatographic strip test [47].

In this study, sixty-six hybridomas secreting monoclonal antibodies were generated and their reactivity and specificity to FMDV were determined by ELISA, western blot and IPMA. The mimotopes recognized by the affinity purified 6F4.D11.B6 and 8D6.B9.C3 were identified by bio-panning with a randomized linear 12-mer phage peptide library. A comparison of the selected phage peptides to the FMDV VP2 amino acid sequences indicated that 6F4.D11.B6 and 8D6.B9.C3 recognized linear epitopes on the VP2 capsid protein located at residues 67–78 and 115–126, respectively. The binding affinity and specificity between VP2 and the phage peptide sequences were confirmed by peptide ELISA. The conclusive results obtained from western blotting, phage ELISA, multiple sequence alignment and peptide ELISA were in accordance and revealed two antigenic epitopes on VP2 of FMDV serotype O.

Thus far, five antigenic sites of FMDV, which are located on the capsid proteins VP1, VP2, and VP3 have been reported [38,48]. Antigenic site 1 is formed by the βG–βH loop (residues 140–160) along with the carboxy terminus of VP1, where amino acid residues 144, 148, 154, and 208 were found to be antigenically critical [38,49]. Antigenic site 2 is located on the βB–βC loop (residues 70–73, 75 and 77) and the adjacent βE–βF loop (residues 131 and 134) of VP2 [50]. Amino acid residues 43, 44, and 45 of the βB–βC loop of VP1 contribute to antigenic site 3 whereas amino acid residues 58 and 60 (βB knob) of VP3 give rise to antigenic site 4. Antigenic site 5 is located at position 149 on the βG–βH loop of VP1. Only site 1 is linear and trypsin-sensitive while the other sites have been reported as conformational epitopes [48].

In addition to antigenic site 2, other epitopes on the VP2 capsid protein of FMDV serotype O were defined based on the sequence analysis of escape mutant FMDVs selected using monoclonal antibodies. The identified neutralizing epitopes of VP2 include residues 68 and 74 of the βB–βC loop, residue 79 adjacent to the βB–βC loop, residues 133 and 134 of the βE–βF loop and residues 188 and 191 of the βH–βI loop [38,48,50,51,52]. Moreover, non-neutralizing conserved epitopes at the N-terminus of VP2, residues 1–5, were also identified and used for the development of a universal diagnostic test for FMDV [53].

In our study, the phage display-derived mimotope ‘HEWNRISDLSYA’ recognized by monoclonal antibody 6F4.D11.B6 was mapped to the residues ^67^FDWVTXDXFGRX^78^ in the BC loop of the VP2 protein of FMDV serotype O which belongs to a part of antigenic site 2. The five known antigenic sites of FMDV mentioned earlier are also identified as neutralizing epitopes [52]. However, 6F4.D11.B6 could not neutralize the viruses although it recognized an epitope located at the antigenic site 2. Many factors affect the efficacy of neutralization, including the stoichiometry of antibody-antigen interaction. Additionally, antibody affinity and the accessibility of the epitopes also play an important role to exceeding the threshold requirement for neutralization [54,55].

Meanwhile, monoclonal antibody 8D6.B9.C3 reacted strongly with FMDV serotype O antigen by ELISA, IPMA, and western blot, and recognized the phage display-derived mimotope ‘SYSGGILSALTE’. This peptide sequence matched with the amino acid positions ^115^QFNGGCLLVAMVP^126^ in the DE loop of VP2, of which the residues 115–124 are highly conserved among serotypes A, Asia I, C, O, and SAT1 [56]. Furthermore, monoclonal antibody 8D6.B9.C3 could neutralize FMDV serotype O suggesting that this epitope is a neutralizing epitope. To our knowledge, this region represents newly identified B cell epitopes within the VP2 protein, although it was previously reported to be a T-cell epitope of FMDV O1 Campos [57]. The findings of this new neutralizing epitope recognized by 8D6.B9.C3 could be used for designing the modern vaccines such as multiple-epitope-vaccines to enhance vaccine efficacy. On the other hand, the epitope recognized by 6F4.D11.B6 might provide cross-protection between FMDV serotypes O and A, co-circulating in Southeast Asia. Moreover, the board-spectrum epitope specific monoclonal antibody is valuable for immunological diagnosis to detect free FMDV particles and intracellular antigens. Additionally, both monoclonal antibodies could bind to free viral particles in the lateral flow immunochromatographic assay developed by our group. These results indicate the value of both monoclonal antibodies for diagnosis, vaccine design and confirmation of FMDV infection in cells and tissues.

## 5. Conclusions

In this study, two monoclonal antibodies, 6F4.D11.B6 and 8D6.B9.C3, were characterized which recognized the epitopes encompassing amino acid residues 67–78 and 115–126 on the capsid protein VP2 of FMDV serotype O, respectively. Additionally, a novel neutralizing epitope on VP2 at residues ^115^QFNGGCLLVAMVP^126^ recognized by 8D6.B9.C3 was identified. Both monoclonal antibodies demonstrated a strong reactivities with various FMDV strains, suggesting their potential application in diagnosis, vaccine designs, and in-depth studies to enhance effective intervention strategy for FMD.

## Figures and Tables

**Table 1:** The sequences of phage clones selected by bio-panning with monoclonal antibodies 6F4.D11.B6 and 8D6.B9.C3 and phage ELISA results.

Monoclonal Antibodies	Amino Acid Sequences	Frequency	Phage ELISA (OD_405_)
6F4.D11.B6	HEWNRISDLSYA	7	3.112
	ATLHSAHRSTHV	2	0.376
8D6.B9.C3	YFPVFPQFNVIQ	1	1.416
	TLHGCCYNSMQR	1	0.733
	FKQDAWEAVDIR	4	0.859
	AISPSRYFYDET	1	1.103
	SYSGGILSALTE	1	0.969

## Data Availability

The original contributions presented in the study are included in the article/Supplementary Materials, further inquiries can be directed to the first author.

## Supplementary Materials

The following supporting information can be downloaded at: https://www.mdpi.com/article/10.3390/antib13030067/s1, Table S1: List of primer sequences for PCR cloning of FMDV serotype O capsid proteins; Table S2: The reactivity of monoclonal antibodies and synthetic peptides in an indirect ELISA; Figure S1: Complete western blotting illustrations of (A) the interaction between 6F4.D11.B6 with FMDV serotypes O and A, (B) the interaction between 8D6.B9.C3 with FMDV serotypes O and A, and (C) the interaction between 6F4.D11.B6 and 8D6.B9.C3 with recombinant FMDV proteins VP1, VP2, and VP3; Figure S2: Alignment of 8D6.B9.C3-specific phage peptide sequences; Figure S3: A visual representation of the MolProbity scores and Ramachandran plots of FMDV capsid protein VP2; Figure S4: A visual representation of the MolProbity scores and Ramachandran plots of monoclonal antibodies 6F4.D11.B6 and 8D6.B9.C3; Figure S5: CDR regions and the predicted binding amino acid residues of 6F4.D11.B6 and 8D6.B9.C3.
