# Supplementary material for: Identification of Conserved Linear Epitopes on Viral Protein 2 of Foot-and-Mouth Disease Virus Serotype O by Monoclonal Antibodies 6F4.D11.B6 and 8D6.B9.C3
Source: Antibodies (Basel). 2024 Aug 7;13(3):67. doi: 10.3390/antib13030067 (PMC11348169; doi:10.3390/antib13030067)
Supplement: Supplementary file 1 [file antibodies-13-00067-s001.zip › antibodies-3041794-supplementary.pdf]

Supplementary data

Table S1 List of primer sequences for PCR cloning of FMDV serotype O capsid proteins.

FMDV proteins	Primer sequences
Serotype O VP1 (OVP1)	F primer: ATGGATCCACCACCTCTGCG<br>R primer: ACAAGCTTTCACTGTTTTGCGGG
Serotype O VP2 (OVP2)	F primer: ATGGATCCGACAAAAAGACG<br>R primer: ATAAGCTTTTACTCCTTGGAAGG
Serotype O VP3 (OVP3)	F primer: ATGGATCCGGGATCTTCCCC<br>R primer: ATAAGCTTTTACTGGGTACGGGC

Table S2 The reactivity of monoclonal antibodies (50 μg/mL) and synthetic peptides (5 μg/mL) in an indirect ELISA. 4F10.D7.F11 is a negative monoclonal antibody control.

mAbs	Peptides	OD 405 nm		
6F4.D11.B6	HEWNRISDLSYA	0.865	0.736	0.638
	unrelated peptide	0.188	0.185	0.147
8D6.B9.C3	SYSGGILSALTE	0.363	0.347	0.329
	unrelated peptide	0.078	0.036	0.038
4F10.D7.F11	HEWNRISDLSYA	0.127	0.113	0.088
	SYSGGILSALTE	0.036	0.023	0.022

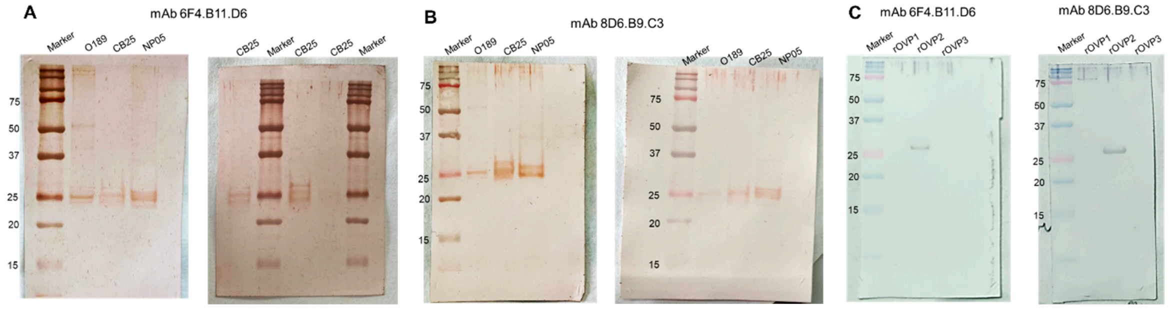

Figure S1 Complete western blotting illustrations of (A) the interaction between 6F4.D11.B6 with FMDV serotype O and A, (B) the interaction between 8D6.B9.C3 with FMDV serotype O and A, and (C) the interaction between 6F4.D11.B6 and 8D6.B9.C3 with recombinant FMDV protein VP1, VP2 and VP3.

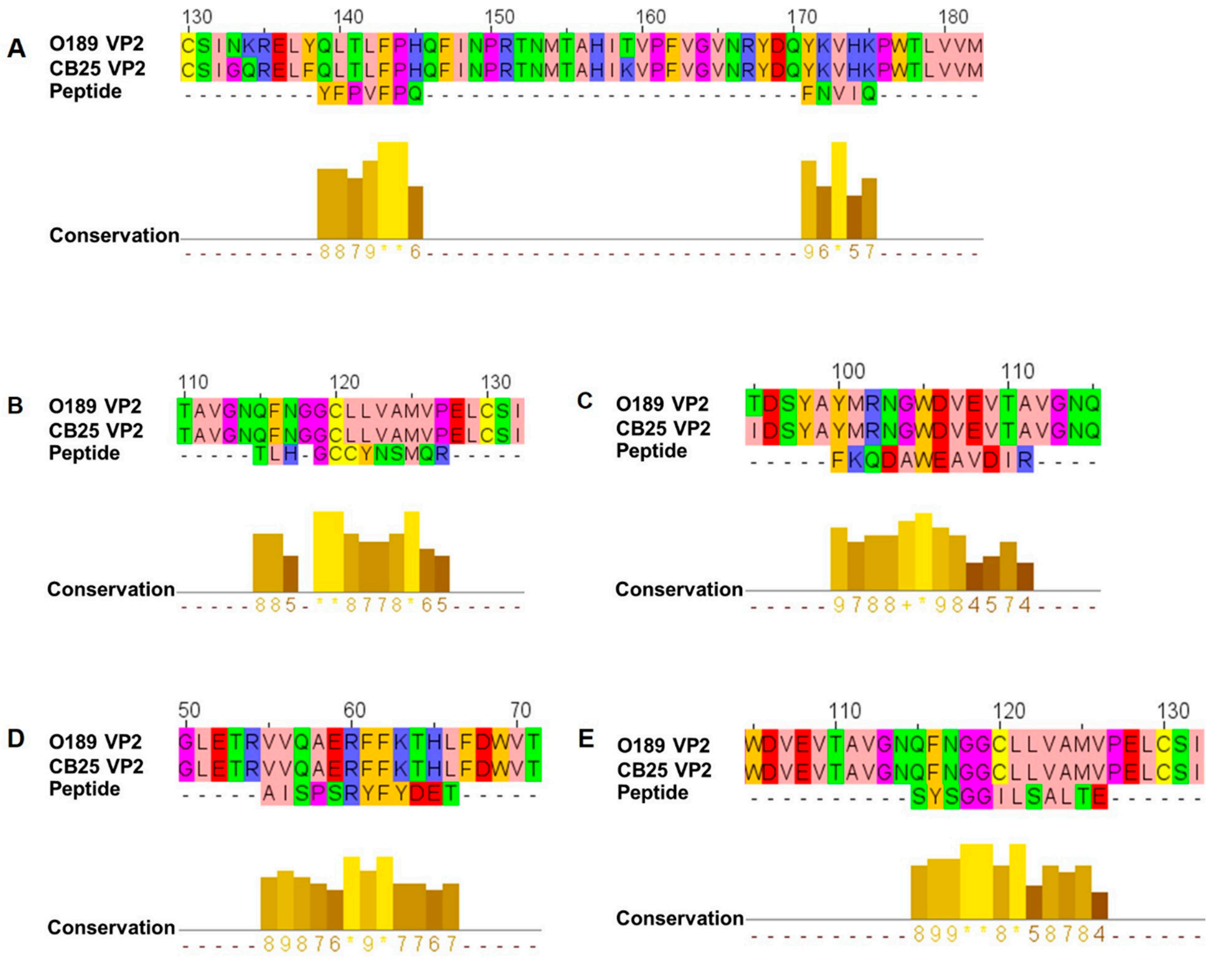

Figure S2 Alignment of 8D6.B9.C3-specific phage peptide sequences: (A) YFPVFPQFNVIQ (B) TLHGCCYNSMQR, (C) FKQDAWEAVDIR, (D) AISPSRYFYDET, (E) SYSGGILSALTE and VP2 amino acid sequence. Each amino acid was colored based on the Zappos scheme, according to their physicochemical properties. Conservation is measured as a numerical index reflecting the conservation of physicochemical properties in the alignment: Identities score highest, and the next most conserved group contain substitutions to amino acids lying in the same physicochemical class. Conservation is visualized on the alignment or a sequence group as a histogram giving the score for each column. Conserved columns are indicated by '*' (score of 11 with default amino acid property grouping), and columns with mutations where all properties are conserved are marked with a '+' (score of 10, indicating all properties are conserved).

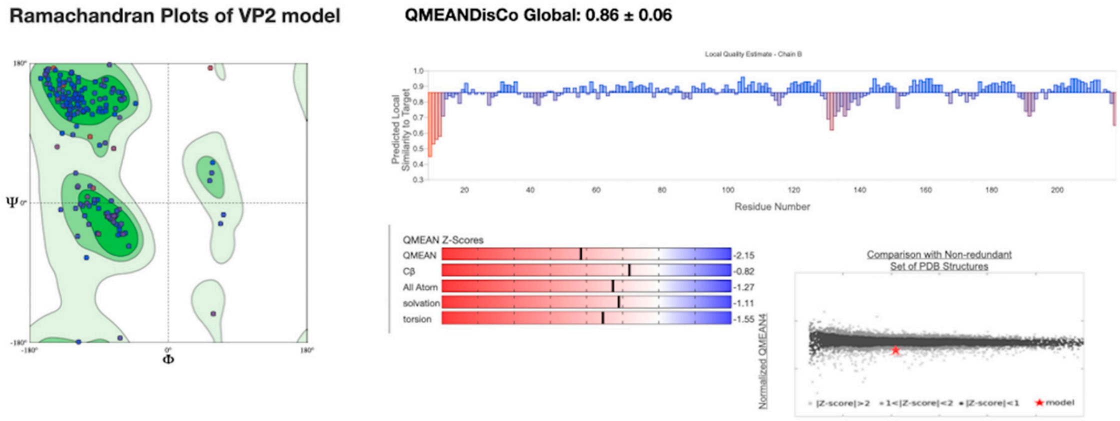

Figure S3 A visual representation of Ramachandran plots and the MolProbity scores of FMDV capsid protein VP2.

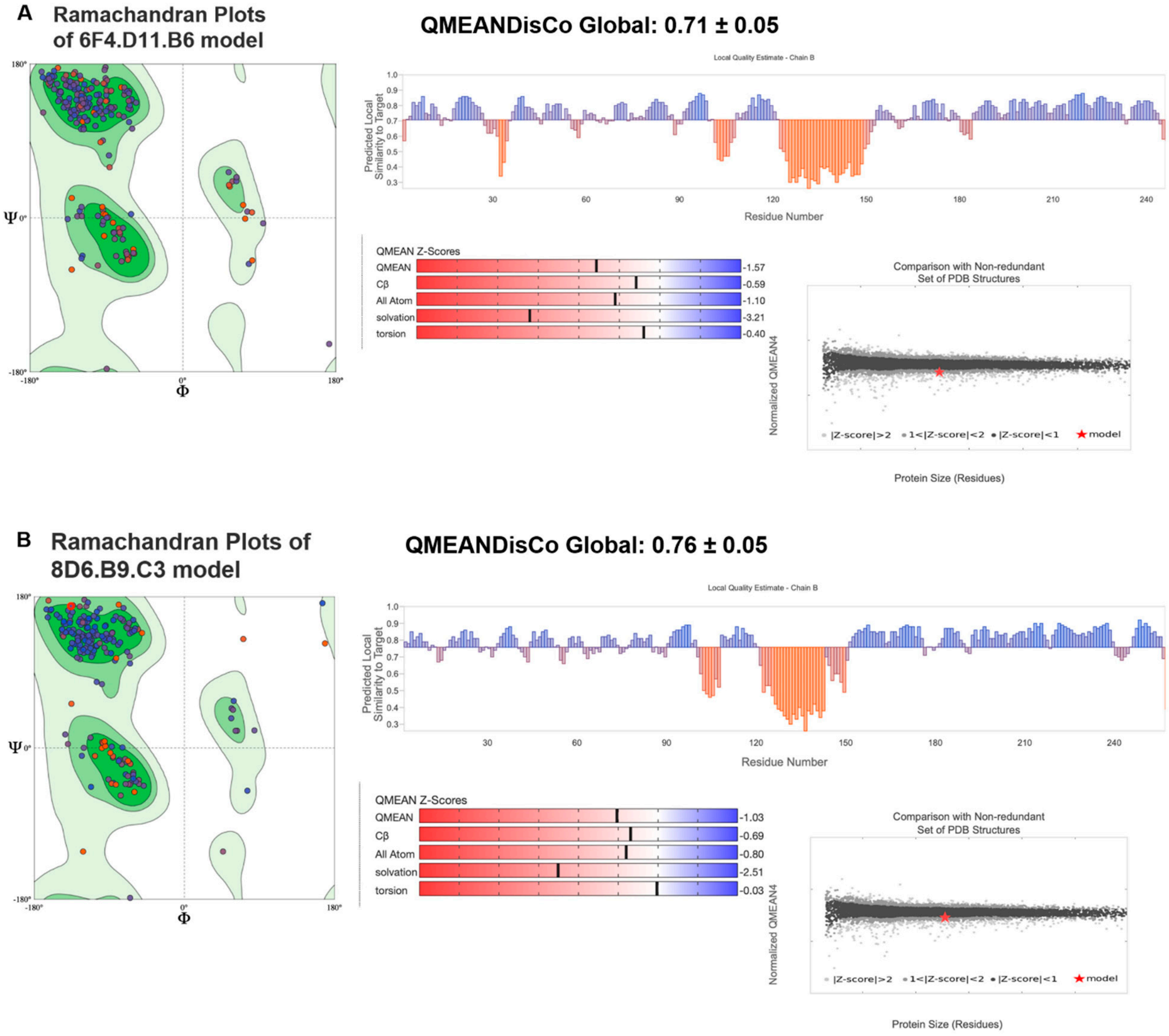

Figure S4 A visual representation of Ramachandran plots and the MolProbity scores of monoclonal antibodies (A) 6F4.D11.B6 and (B) 8D6.B9.C3.

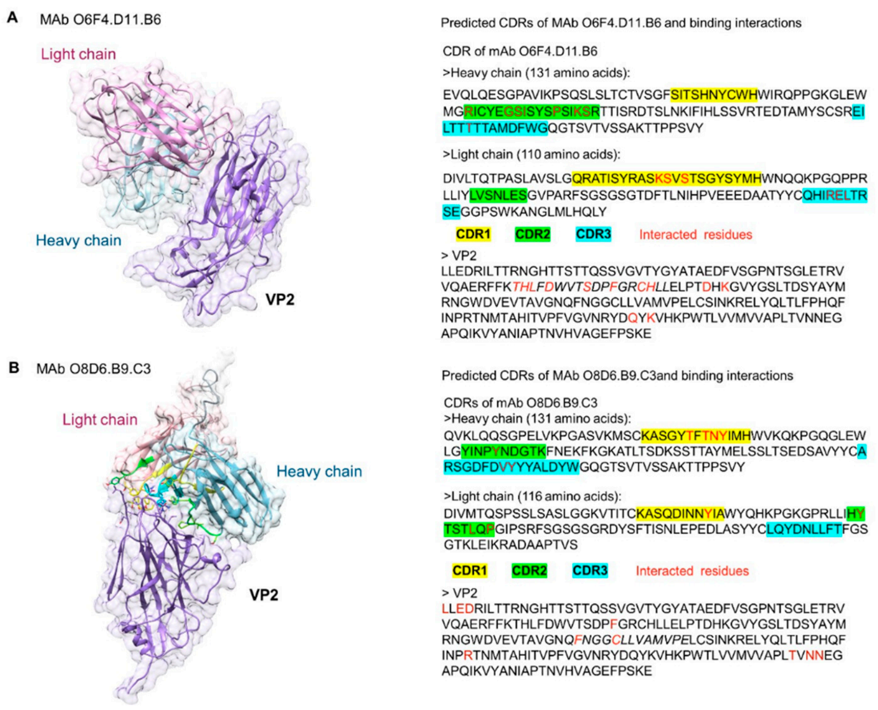

Figure S5 CDR regions and the predicted binding amino acid residues of (A) 6F4.D11.B6 and (B) 8D6.B9.C3. The molecular docking analysis was conducted using HADDOCK to explore the binding interactions between FMDV-specific monoclonal antibodies and FMDV serotype O capsid protein VP2. Both monoclonal antibodies showed the different interactions with the surface-exposed loops of FMDV serotype O VP2 capsid protein. The models were visualized and analyzed using Discovery Studio Visualizer, version 2021 (BIOVIA, Dassault Systèmes, San Diego, CA, USA), and UCSF Chimera, version 1.16 (UCSF, San Francisco, CA, USA).
